# Relationship of sleep-quality and social-anxiety in patients with breast cancer: a network analysis

**DOI:** 10.1186/s12888-023-05262-1

**Published:** 2023-11-28

**Authors:** Chunyan He, Yang He, Tianqi Yang, Chao Wu, Yawei Lin, Jiaran Yan, Wei Chang, Fenxia Chang, Yameng Wang, Shengjun Wu, Baohua Cao

**Affiliations:** 1 Department of Nursing, Fourth Military Medical University, Xi’an, 710032 Shaanxi China; 2 Department of Psychology, Fourth Military Medical University, Xi’an, 710032 Shaanxi China; 3 Center for Aerospace Clinical Medicine, Department of Aerospace Medicine, Fourth Military Medical University, Xi’an, 710032 Shaanxi China; 4 Department of Obstetrics and Gynecology, Xi ’an Daxing Hospital, Xi’an, 710000 Shaanxi China; 5 School of Clinical Medicine, Anhui Medical University, Hefei, 230000 Anhui China

**Keywords:** Network analysis, Social anxiety, Sleep quality, Breast cancer

## Abstract

**Background:**

There is a complex relationship between social anxiety and sleep quality. However, network analysis studies of associations between social anxiety and sleep quality are lacking, particularly among patients with breast cancer. The current study aimed to extend this research to a sample of patients with breast cancer and to examine symptom-level associations between social anxiety and sleep quality using network analysis.

**Methods:**

Network analysis was conducted to explore their associations and identify bridge items of social anxiety and sleep quality.

**Results:**

The network structure revealed 9 important edges between social anxiety and sleep quality. “Subjective sleep quality” had the highest EI value in the network. “Working difficulty under watching” and “Sleep disorders” had the highest BEI values in their own communities.

**Conclusion:**

There are complex pathological correlation pathways between social anxiety and sleep quality in breast cancer patients. “Subjective sleep quality”, “Working difficulty under watching” and “Sleep disorders” have the potential to be intervention targets for sleep disorder-social anxiety comorbidity. Medical staff can take corresponding interventions according to the the centrality indices and bridge centrality indicators identified in this study, which is likely to effectively reduce the comorbidity of sleep disorders and social anxiety.

**Supplementary Information:**

The online version contains supplementary material available at 10.1186/s12888-023-05262-1.

## Background

Breast cancer has become the most common type of new cancer worldwide and ranks first in the incidence of malignant tumors in women in China [1]. At present, China’s clinics usually use radiotherapy, chemotherapy and surgical excision-based comprehensive treatment plans for breast cancer [2]. A variety of treatment methods significantly improves the survival rate of cancer patients but does change the body image of women, resulting in patients having adverse emotional reactions [3]. Social anxiety is an emotional experience characterized by nervousness and fear that individuals experience in social situations where they come into contact with or are evaluated by others [4]. Due to the absence of the postoperative breast and scarring of the surgical site, some breast cancer patients no longer want to look straight at their bodies, feeling a decrease in external attractiveness, and developing a strong sense of inferiority and a heavy psychological burden [5], leading to a decrease in self-esteem levels, fear of looking at and being looked at by others, avoidance of interaction with others, and even reluctance to participate in social activities [6]. Relevant studies show that breast cancer patients have more severe social anxiety after surgery [7], and all social interactions are affected to different degrees. Therefore, exploring the mechanisms of social anxiety with a view to finding targets for early intervention is of great clinical and practical importance for improving normal social interactions in breast cancer patients.

Sleep disturbance is a common symptom in breast cancer patients [8]. Studies have shown that the incidence of sleep disturbance in cancer patients ranges from approximately 30–75% [9], while the incidence of sleep disturbance in postoperative breast cancer patients is surprisingly high at more than 90% [10]. Kakizaki et al [11]. found that poorer sleep quality was not only a risk factor for breast cancer development but also an important factor in the reduced quality of life of patients. Sleep is a key biological behavior for maintaining the immune and endocrine functions of the body [12] and plays a crucial role in mood and cognitive behavior [13]. Studies have shown that sleep quality affects the hypothalamic‒pituitary‒adrenal axis (HPA) and the sympathetic nervous system (SNS), and that poor sleep quality synergistically increases the proinflammatory effects of both systems [14], and causing physiological discomfort to patients [15].Therefore, it is important to elucidate the factors that affect the sleep quality of breast cancer patients in order to improve their quality of life and ultimately their prognosis.

Social anxiety and sleep disturbance, as the two major risk symptoms of breast cancer patients, are closely related to each other. Social anxiety has been reported to affect the cardiovascular system, neuroendocrine system, and cognitive function in humans and even leads to cognitive decline and sleep problems [16]. In turn, previous studies have also suggested that physical discomfort, symptom distress, fear, anxiety, and depression in cancer patients are positively associated with even their sleep quality [17]. To test the theoretical link between social anxiety and sleep quality, Simon and Walker [18] found that sleep-deprived participants was more likely to become wary and hostile to those around us and thus reject social interaction; Bach et al [19]. have shown that Sleep quality is closely related to social emotions, and the two can affect each other, which in turn affects the postoperative recovery of breast cancer patients.

However, previous studies on social anxiety and sleep quality suffer from two major shortcomings. One is the use of a quantitative approach that tends to factor in social anxiety and sleep quality based on the total score of a scale, i.e., to assess their correlation by the overall score. In fact, total scale scores based on item equivalence ignore the heterogeneity of the symptoms represented by the different items, which actually have different weightings in the occurrence of the problem. Second, some findings are mainly based on behavioral studies, that is, studies that highlight a behavioral performance-related interconnection of items and then allow speculation on the internal mechanisms of association between the items. However, both quantitative and behavioral studies lack a fine-grained understanding of the interrelationship [20]. In contrast, network analysis can clarify the fine-grained relationships between scale items [22] and help us find the best targets for effective interventions by correlating individual dimensions and entries in a way that has not been done before. Therefore, it is necessary to explore the patterns of association between different items of social anxiety and different dimensions of sleep quality.

Network analysis is a promising statistical method and network structure consists of nodes representing psychological variables and connecting lines representing statistical relationships between variables [23]. Compared to traditional statistical models, network analysis has three advantages: first, it can clarify the fine-grained relationships between individual variables [24]; second, it can visualize the interactions between variables [25]; and third, it can evaluate the relative importance of different nodes that are interrelated in the network by computing indicators [24]. Thus, in this study, network analysis helped us to compare the role of different factors of social anxiety on different dimensions of sleep quality in breast cancer patients.

In summary, this study used network analysis to investigate the fine-grained relationship between social anxiety and sleep quality in breast cancer patients, thus providing theoretical support for the prevention and intervention of social anxiety and sleep quality in patients with breast cancer.

## Methods

### Participants

In this cross-sectional study, postoperative patients with breast cancer who came from three tertiary grade-A hospitals in Xi’an, China, between May 2021 and October 2021 were considered for participation. The patients were recruited for the research via the convenience sampling method and met the following inclusion criteria: (1) diagnosed with breast cancer based on pathology;(2) were female; (3) were aged 18 ~ 60; (4) of clear mind without cognitive impairment or communication impairment; and (5) gave informed consent and volunteered to participate in the research. The exclusion criteria were as follows: (1) recurrence or metastasis of breast cancer during treatment; (2) other malignancies and significant organ dysfunction; and (3) a previous history or family history of severe mental illness.

A rough estimation method of sample size was used: 5–10 times the number of study variables was used to calculate the sample size [26]. There were 22 variables in the study; hence, the sample size of this study was 110–220 patients. Considering a loss rate of 20%, the minimum sample size of the study was 132 patients.

### Data collection

The researchers explained the purpose, significance and content of the study to patients who met the inclusion criteria and obtained their informed consent. Participants completed a paper-based questionnaire anonymously using standard guidelines and were assured of the confidentiality of all research data.

### Measurements

#### The Chinese version of the social anxiety scale

The Chinese version of the Social Anxiety Scale (C-SAS) translated into Mandarin Chinese by Wang [27] includes 6 items. This study used abbreviations to denote the meaning of each item. For example, “Shyness in new environment” means “It took me a long time to get over my shyness in my new environment.” Each item is rated on a 5-point Likert scale (0 = “very inconsistent” to 4 = “very consistent,” and the total score ranges from 0 to 24 points. Higher scores indicate that patients experience social anxiety to a greater degree. The questionnaire in the present study had excellent internal consistency (Cronbach’s α = 0.732).

#### Pittsburgh sleep quality index scale (PSQI)

The Pittsburgh Sleep Quality Index Scale (PSQI) developed by Buysse et al [28]. consists of 18 items with 7 dimensions, which are subjective sleep quality, sleep latency, sleep duration, habitual sleep efficiency, sleep disturbance, hypnotic drugs, and daytime dysfunction. Each dimension is scored on a scale of 0 to 3, with a total score ranging from 0 to 21 points. Higher scores indicate worse sleep quality. The questionnaire in the present study had excellent internal consistency (Cronbach’s α = 0.828).

### Data analysis

All descriptive data were analyzed using SPSS 25.0 software. Network analysis was used R 4.1.1 software.

#### Network analysis

##### Network building and evaluation

We used the qgraph and bootnet R packages [29] to build and evaluate the social anxiety-sleep quality network in patients with breast cancer. In the network, the blue and red lines represent positive and negative correlations between symptoms of social anxiety and sleep quality, respectively, and the thickness of the line and the saturation of the color represent the magnitude of the correlation [30]. The nodes in the network are divided into two communities according to their sources, that is, the social anxiety community and the sleep quality community. The least absolute shrinkage and selection operator (LASSO) [31] regularization and extended Bayesian information criterion (EBIC) [32] can shrink all edges and make meaningless edges zero weight [30], thus building a stable and comprehensible network. We set the EBIC hyperparameter to 0.5 and used Spearman correlation.

We used the nonparametric bootstrapping method to evaluate the accuracy of the edge weight (1000 bootstrapped samples), and the good accuracy of the edge weight was represented by a narrow 95% confidence interval [30]. We used the bootstrapping method to conduct the difference test of edge weights of different edges (1000 bootstrapped samples, α = 0.05).

##### Centrality and bridge centrality calculation and evaluation

We used the networktools and bootnet R packages [30] to calculate and evaluate the expected influence (EI) and bridge expected influence (BEI) of nodes. The EI of a node refers to the sum of the edge weights between the node and all the other nodes in the network and reflects the importance of the node in the network [34]. The BEI of a node refers to the sum of edge weights between the node and all the nodes from other communities and reflects the impact on other communities [33]. We used the case-dropping bootstrapping method to test the stability of EI and BEI (1000 bootstrapped samples). Then, we used the correlation stability (CS) coefficient to quantify the stability of EI and BEI; generally, acceptable stability is represented by a CS coefficient greater than 0.25. We used the bootstrapping method to conduct the difference tests of EIs and BEIs of different nodes (1000 bootstrapped samples, α = 0.05).

## Results

### Demographic characteristics and descriptive statistics

A total of 300 questionnaires were collected. Finally, 293 questionnaires were received, for an effective response rate of 97.7%. The demographic characteristics of the participants are displayed in Table 1. The mean scores, standard deviations, EIs and BEIs of items in the social anxiety-sleep quality network are displayed in Table 2.

**Table 1:** The demographic characteristics of participants (n = 293)

Variables	Number(n)	Percentage (%)
**Age (years)**		
20–30	13	4.4
31–40	72	24.6
41–50	109	37.2
51–60	99	33.8
**Educational level**		
Junior secondary and below	106	36.2
High school/junior college	113	38.6
Bachelor and above	74	25.2
**Occupation**		
Enterprises/institutions	93	31.7
Unemployed	88	30.0
Others	112	38.3
**Marital status**		
Married	273	93.2
Unmarried/divorced/widowed	20	6.8
**Family monthly income per capita (RMB)**		
< 3000	124	42.4
3000 ~ 5000	100	34.1
> 5000	69	23.5
**Place of residence**		
Urban area	214	73.0
Rural area	79	27.0
**Live alone**		
Yes	21	7.2
No	272	92.8
**Treatment stage**		
Surgery	110	37.5
Chemotherapy	100	34.1
Radiotherapy	33	11.3
Other	50	17.1
**Surgical method**		
Breast-conserving	211	72.0
Radical mastectomy	82	28.0

**Table 2:** The mean scores, standard deviations, EIs and BEIs of items in the social anxiety-sleep quality network

Items	Abbreviations / Dimensions	M	SD	EI	BEI
**Social Anxiety**					
A1: It took me a long time to get over my shyness in my new environment	Shyness in new environment	1.57	1.15	0.57	0.00
A2: I have a hard time working when someone is watching me	Working difficulty under watching	1.30	1.16	0.91	0.19
A3: I get sleepy very easily	Getting sleepy easily	1.81	1.24	0.41	0.01
A4: It is hard for me to talk to strangers	Talking to strangers difficultly	1.62	1.11	0.19	0.00
A5: I get nervous talking in front of a crowd	Talking nervously in front of a crowd	1.54	1.18	0.93	0.04
A6: Being in a large group of people makes me nervous	Being nervous in a large group	1.45	1.21	0.90	0.04
**Sleep quality**					
S1: Subjective sleep quality	Subjective sleep quality	1.32	0.81	1.24	0.04
S2: Sleep latency	Sleep latency	1.44	1.20	0.99	0.03
S3: Sleep duration	Sleep duration	1.27	0.94	0.63	0.00
S4: Habitual sleep efficiency	Habitual sleep efficiency	1.16	0.94	1.02	0.03
S5: Sleep disturbance	Sleep disturbance	1.29	0.51	0.48	0.12
S6: Hypnotic drugs	Hypnotic drugs	0.14	0.51	0.23	− 0.01
S7: Daytime dysfunction	Daytime dysfunction	1.79	1.07	0.45	0.07

### Network model

**Fig. 1:**
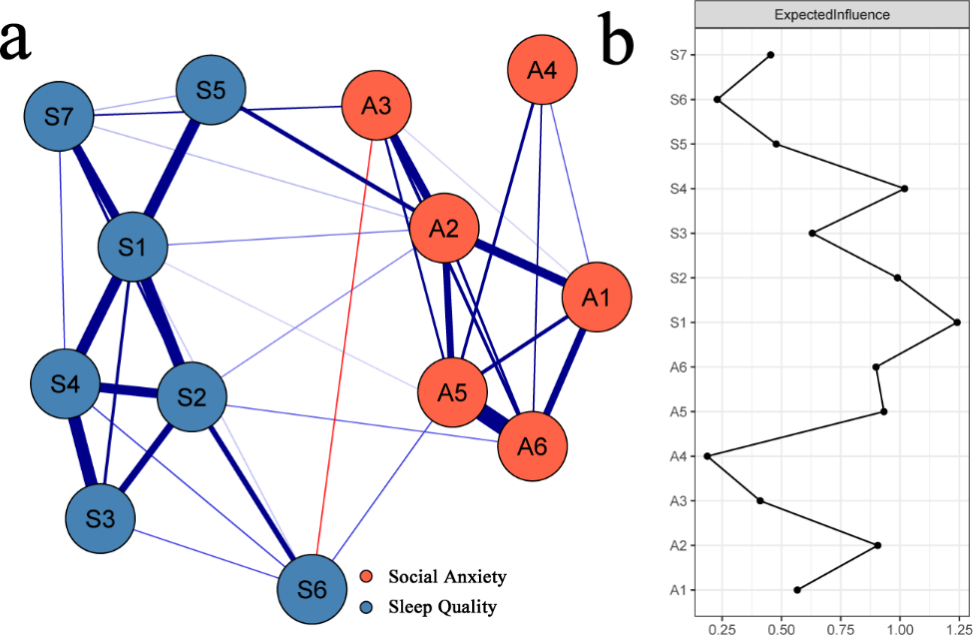
The social anxiety-sleep quality network model in breast cancer patients and EIs of the nodes in the network. Note: **(a)** The social anxiety-sleep quality network model in breast cancer patients. **(b)** EIs of the nodes in the network (raw scores). The blue and red lines represent positive and negative correlations between symptoms of social anxiety and sleep quality, and the thickness of the line and the saturation of the color represent the magnitude of the correlation. A1 = shyness in new environment; A2 = working difficulty while watching; A3 = getting sleepy easily; A4 = talking to strangers difficultly; A5 = talking nervously in front of a crowd; A6 = being nervous in a large group; S1 = subjective sleep quality; S2 = sleep latency; S3 = sleep duration; S4 = habitual sleep efficiency; S5 = sleep disturbance; S6 = hypnotic drugs; S7 = daytime dysfunction

The social anxiety-sleep quality network model is displayed in Fig. 1a. There are 9 nonzero edges across the communities (weight range from − 0.05 to 0.12). A2 “working difficulty under watching” was correlated with 4 nodes from the sleep quality community, namely, S1 “subjective sleep quality,” S2 “sleep latency,” S5 “sleep disturbance” and S7 “daytime dysfunction,” and the strongest correlation was with S5 “sleep disturbance” (edge weight = 0.12). A3 “getting sleepy easily” was correlated with 2 nodes from the sleep quality community, namely, S6 “hypnotic drugs” and S7 “daytime dysfunction,” and the strongest correlation was with S7 “daytime dysfunction” (edge weight = 0.06). A5 “talking nervously in front of a crowd” was correlated with 1 node from the sleep quality community, namely, S6 “hypnotic drugs” (edge weight = 0.04). A6 “being nervous in a large group” was correlated with 2 nodes from the sleep quality community, namely, S1 “subjective sleep quality” and S4 “habitual sleep efficiency,” and the strongest correlation was with S4 “habitual sleep efficiency” (edge weight = 0.03). A1 “shyness in new environment” and A4 “talking to strangers difficultly” were not directly correlated with the nodes from the sleep quality community. The correlation matrix among nodes in the network can be found in Table S1 of the supplementary material.

As displayed in Figure S1 in the supplementary material, the 95% confidence interval of edge weights was relatively narrow in the social anxiety-sleep quality network, indicating acceptable accuracy. The result of the difference test of the edge weights is displayed in Figure S2 in the supplementary material.

### Central symptoms

As displayed in Fig. 1b, S1 “subjective sleep quality” showed the highest EI value (EI = 1.24) and was regarded as the central symptom in the social anxiety-sleep quality network. The results of the stability test of EIs are displayed in Figure S3 in the supplementary material. The CS coefficient for EIs was 0.672, which indicated ideal stability. The results of the difference test of EIs are displayed in Figure S4 in the supplementary material. The EI value of S1 “subjective sleep quality” was significantly larger than that of all the other nodes in the network.

### Bridge symptoms

**Fig. 2:**
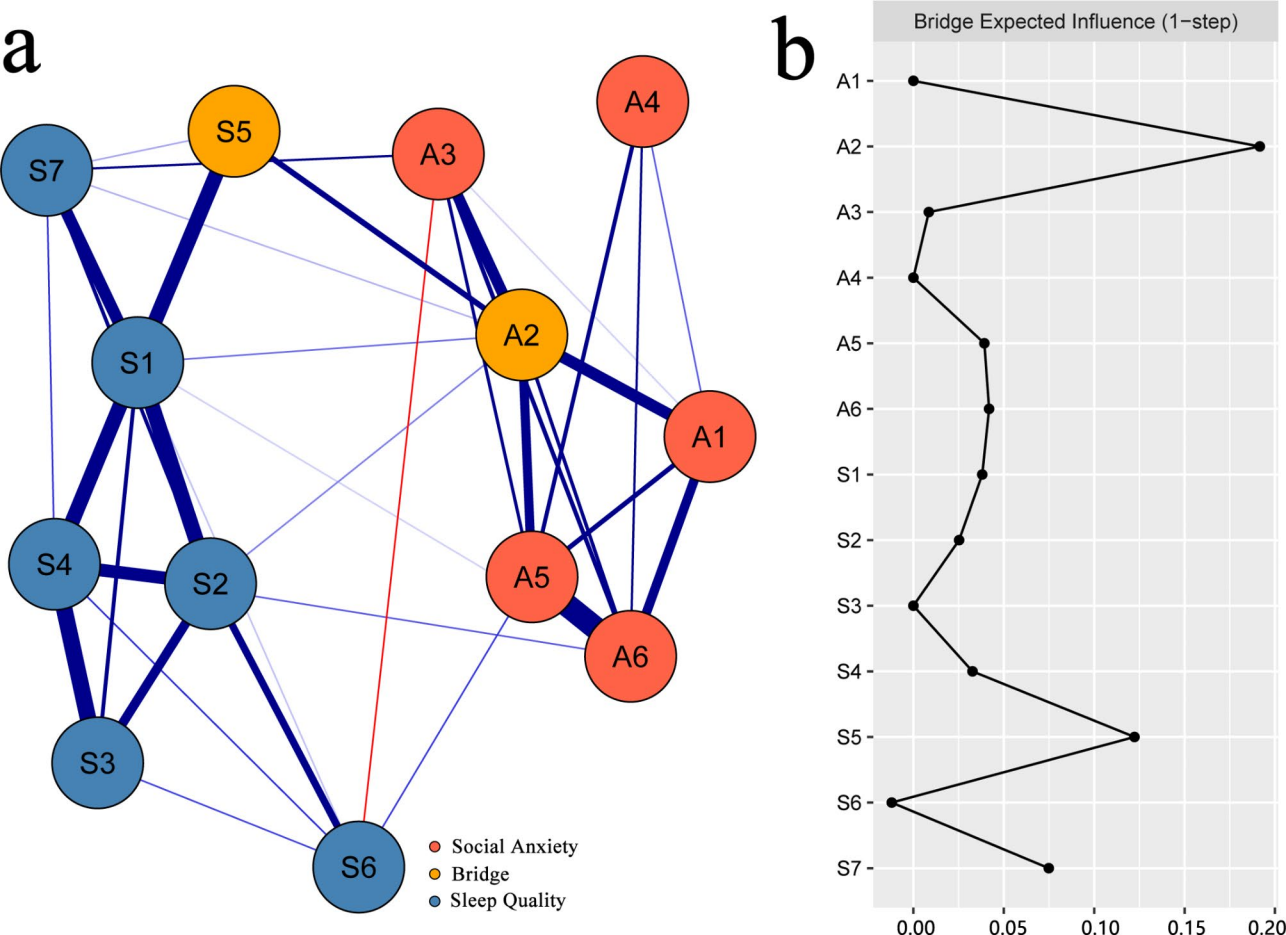
The bridge symptoms in the social anxiety-sleep quality network of breast cancer patients and BEIs of the nodes in the network. Note: **(a)** The bridge symptoms in the social anxiety-sleep quality network of breast cancer patients. **(b)** The BEIs of nodes in the network (raw scores). The blue and red lines represent positive and negative correlations between symptoms of social anxiety and sleep quality, and the thickness of the line and the saturation of the color represent the magnitude of the correlation. A1 = shyness in new environment; A2 = working difficulty while watching; A3 = getting sleepy easily; A4 = talking to strangers difficultly; A5 = talking nervously in front of a crowd; A6 = being nervous in a large group; S1 = subjective sleep quality; S2 = sleep latency; S3 = sleep duration; S4 = habitual sleep efficiency; S5 = sleep disturbance; S6 = hypnotic drugs; S7 = daytime dysfunction

As shown in Fig. 2a, the BEIs of nodes in the social anxiety-sleep quality network are displayed in Fig. 2b. A2 “working difficulty under watching” and S5 “sleep disturbance” showed the highest BEI values in their own community (BEI = 0.19, 0.12). Therefore, A2 “working difficulty under watching” and S5 “sleep disturbance” were regarded as bridge symptoms in the social anxiety-sleep quality network. The results of the stability test of BEIs are displayed in Figure S5 in the supplementary material. The CS coefficient for BEIs was 0.283, which indicated acceptable stability. The result of the difference test of BEIs is displayed in Figure S6 in the supplementary material.

## Discussion

In this study, we explored the structure of social anxiety and sleep quality in a group of patients with breast cancer using network analysis. To our knowledge, this is the first network analytic study of the comorbidity between these two constructs. The detailed relationships between the variables revealed by network analysis may provide an indication of the relevant pathways by which social anxiety and sleep quality interact [35].

Among the four pathways through which A2“working difficulty under watching” correlated with sleep quality, the strongest pathway was A2 “working difficulty under watching”—S5“sleep disturbance.” “Working difficulty under watching,” a classic form of social anxiety, may arise mainly because individuals care too much about what others think and evaluate [37]. The reason may be that breast cancer patients, because of disease and image problems in social and work processes, often have low self-esteem and are sensitive to negative emotions [38]. Therefore, the internalization of these negative feelings may lead to a disruption of the sleep-wake cycle [39]. Sleep disturbance mainly refers to the amount of abnormal sleep; abnormal behavior, including night terrors and nightmares [40], often occurs during sleep and is often accompanied by anxiety, depression and other negative emotions [41]. Interestingly, A2 “working difficulty under watching” and S5 “sleep disturbance” showed the highest BEI values in their own community. This means that A2 “working difficulty under watching” has the greatest impact on sleep quality community, and S5 “sleep disturbance” has the greatest impact on social anxiety community. Statistically, we can minimize the probability of comorbidity between social anxiety and sleep disorders if we intervene to cut them off [33]. Of course, this needs to be tested through practice.

Between the two pathways through which A3 “getting sleepy easily” correlated with sleep quality, the stronger pathway was A3 “getting sleepy easily”—S7 “daytime dysfunction.” Patients with breast cancer often experience cancer-related fatigue, drowsiness, and low energy due to the effects of disease and treatment, which may affect their ability to cope with social interactions [42]. Excessive daytime sleepiness, drowsiness, and work energy deficiency are the typical manifestations of daytime dysfunction [43]. In addition, insufficient sleep can lead to the development and maintenance of cancer therapy-related fatigue [44]. Therefore, the correlation between these two symptoms is not difficult to understand. In addition, we found an interesting negative correlation between A3 “getting sleepy easily” and S6 “hypnotic drugs,” whereas previous studies have found a positive correlation between social anxiety and poor sleep quality [45]. The reason may be that previous research often used the total score to do correlation, hiding the correlation within the two communities [46]. This study found that A5 “talking nervously in front of a crowd” was positively correlated with S6 “hypnotic drugs.” A possible reason is that patients with breast cancer have low self-esteem, which can lead to social anxiety. The serious anxious mood can make it difficult for individuals to fall asleep [48]. Therefore, some patients may take hypnotic drugs to improve sleep. In addition, among the two pathways through which A6 “being nervous in a large group” correlated with sleep quality, the stronger pathway was A6 “being nervous in a large group”-S4 “habitual sleep efficiency.” Breast cancer patients who fear the visibility of their disease may draw negative attention in social situations [49], which may contribute to social anxiety and social withdrawal. Sleep efficiency is defined as the ratio of the total sleep time in a night compared with the total time spent in bed [50]. One study showed that individuals with low self-esteem and anxiety have lower sleep efficiency [51], similar to the results of this study. The possible reason is that when an individual is in a state of anxiety, he or she will often have difficulty falling asleep, and his or her sleep efficiency will decrease accordingly.

Among all the symptoms included in this network, the sleep quality symptom S1 “subjective sleep quality” had the highest EI centrality. The high EI centrality indicated that targeting this node may provide the highest general benefit to other nodes in the network [52]. For patients with both social anxiety and sleep quality, targeting this symptom may provide the highest benefit. Subjective sleep quality refers to the subjective judgment of the patient according to their own sleep condition [53]. Poor subjective sleep quality is the patient’s subjective dissatisfaction with the duration and quality of sleep [53]. Studies have shown that individuals’ feelings and evaluations of their own sleep affect their level of concern about sleep disorders and their willingness to treat them [54]. Among them, individuals with poor subjective sleep quality are more likely to be concerned about sleep disorders, and they tend to feel fatigue, poor concentration, and depressed mood, which affect normal daytime functioning and physiological status [55], thus further aggravating anxiety states [56], and even affecting normal socialization and leading to social alienation [57]. Therefore, in assessing, preventing, and improving the co-morbidity of sleep disorders and social anxiety, it is may need first concentrate on how to improve subjective sleep quality with less focus on other symptoms. It is of great significance in preventing social anxiety, improving the prognosis of diseases and reducing the waste of medical resources. The main intervention strategies on sleep in breast cancer patients include drug therapy and nondrug therapy. Drug therapy is a common therapy for clinical sleep disorder. The common nondrug therapies include cognitive behavioral therapy (CBT) [58] and exercise therapy [59]. CBT includes sleep restriction therapy, relaxation training therapy and cognitive therapy and is thought to be the best intervention for chronic insomnia [60]. Rogers et al [61]. found that aerobic exercise (swimming, calisthenics, walking, etc.) in exercise therapy also significantly reduced fatigue and sleepiness and improved sleep quality. Other studies have shown that mindfulness-based interventions [62] and bright light therapy [63] play important roles in improving sleep quality in breast cancer patients. Therefore, medical staff should carefully assess the patient’s sleep and adopt appropriate methods to improve their sleep quality.

This original study provides a new direction for intervention in the comorbidity of social anxiety with sleep disorders in breast cancer patients. however, this study has some limitations worth mentioning. First, the study included only female patients with breast cancer. These findings may not be generalizable to survivors of other types of cancer. Second, our study relied solely on self-reported psychometric data without clinical diagnosis for symptom assessment. Future studies could validate our findings on the basis of the same symptoms identified by a professional clinical diagnosis. In addition, the network structure constructed here investigated the effects among the variables based on the population level. This means that within a single individual, the network structure may not be replicated in the same way.

## Conclusions

In summary, this study is the first to investigate the network structure of social anxiety and sleep quality in female patients with breast cancer. For centrality indices, “subjective sleep quality” had the highest EI. “Working difficulty under watching” and “sleep disturbance” are key to understanding sleep quality with comorbid social anxiety disorders in the network model presented here. Future studies should try to verify the intervention targets suggested in this study and explain this bridging function more precisely with clinical practice or suitable methods beyond psychometric questionnaires.

### Electronic supplementary material

Below is the link to the electronic supplementary material.

Supplementary Material 1: **Figure S1**. Accuracy test of edge weights in the social anxiety-sleep quality network. Note: The gray area represents the bootstrapped confidence intervals and the red line represents the sample edge weight values. A1 = shyness in new environment; A2 = working difficulty while watching; A3 = getting sleepy easily; A4 = talking to strangers difficultly; A5 = talking nervously in front of a crowd; A6 = being nervous in a large group; S1 = subjective sleep quality; S2 = sleep latency; S3 = sleep duration; S4 = habitual sleep efficiency; S5 = sleep disturbance; S6 = hypnotic drugs; S7 = daytime dysfunction

Supplementary Material 2: **Figure S2**. Bootstrapped difference test of edge weights in the social anxiety-sleep quality network. Note: The gray box represents that the edge weights of the two corresponding node pairs have no significant difference, and the black box represents a significant difference. Blue and red boxes on the diagonal represent positive and negative edge weights, respectively. A1 = shyness in new environment; A2 = working difficulty while watching; A3 = getting sleepy easily; A4 = talking to strangers difficultly; A5 = talking nervously in front of a crowd; A6 = being nervous in a large group; S1 = subjective sleep quality; S2 = sleep latency; S3 = sleep duration; S4 = habitual sleep efficiency; S5 = sleep disturbance; S6 = hypnotic drugs; S7 = daytime dysfunction

Supplementary Material 3: **Figure S3**. Stability of expected influences in the social anxiety-sleep quality network. Note: The red bar represents the average correlation between expected influences in the full sample and subsample with the red area depicting the 2.5th quantile to the 97.5th quantile

Supplementary Material 4: **Figure S4**. Bootstrapped difference test of expected influences in the social anxiety-sleep quality network. Note: The black box indicates that the expected influences of the two corresponding nodes have a significant difference, the gray box indicated no significant difference. A1 = shyness in new environment; A2 = working difficulty while watching; A3 = getting sleepy easily; A4 = talking to strangers difficultly; A5 = talking nervously in front of a crowd; A6 = being nervous in a large group; S1 = subjective sleep quality; S2 = sleep latency; S3 = sleep duration; S4 = habitual sleep efficiency; S5 = sleep disturbance; S6 = hypnotic drugs; S7 = daytime dysfunction

Supplementary Material 5: **Figure S5**. Stability of bridge expected influences in the social anxiety-sleep quality network. Note: The red bar represents the average correlation between bridge expected influences in the full sample and subsample with the red area depicting the 2.5th quantile to the 97.5th quantile.

Supplementary Material 6: **Figure S6**. Bootstrapped difference test of bridge expected influences in the social anxiety-sleep quality network. Note: The black box indicates that the bridge expected influences of the two corresponding nodes have a significant difference, the gray box indicated no significant difference. A1 = shyness in new environment; A2 = working difficulty while watching; A3 = getting sleepy easily; A4 = talking to strangers difficultly; A5 = talking nervously in front of a crowd; A6 = being nervous in a large group; S1 = subjective sleep quality; S2 = sleep latency; S3 = sleep duration; S4 = habitual sleep efficiency; S5 = sleep disturbance; S6 = hypnotic drugs; S7 = daytime dysfunction.

Supplementary Material 7: **Table S1**. The correlation matrix in the social anxiety-sleep quality network of breast cancer patients

## Data Availability

The raw data supporting the conclusions of this article will be made available by the authors without undue reservation. Please contact the corresponding author.

## Abbreviations

C-SAS: The Chinese version of the social anxiety scale
PSQI: The pittsburgh sleep quality index scale
Shyness in new environment: It took me a long time to get over my shyness in my new environment
Working difficulty under watching: I have a hard time working when someone is watching me
Getting sleepy easily: I get sleepy very easily
Talking to strangers difficultly: It is hard for me to talk to strangers
Talking nervously in front of a crowd: I get nervous talking in front of a crowd
Being nervous in a large group: Being in a large group of people makes me nervous
LASSO: Least absolute shrinkage and selection operator
EBIC: Extended bayesian information criterion
EI: Expected influence
BEI: Bridge expected influence
CS: Correlation stability
