# Supplementary figures and images for: Relationship of sleep-quality and social-anxiety in patients with breast cancer: a network analysis
Source: BMC Psychiatry. 2023 Nov 28;23:887. doi: 10.1186/s12888-023-05262-1 (PMC10683122; doi:10.1186/s12888-023-05262-1)

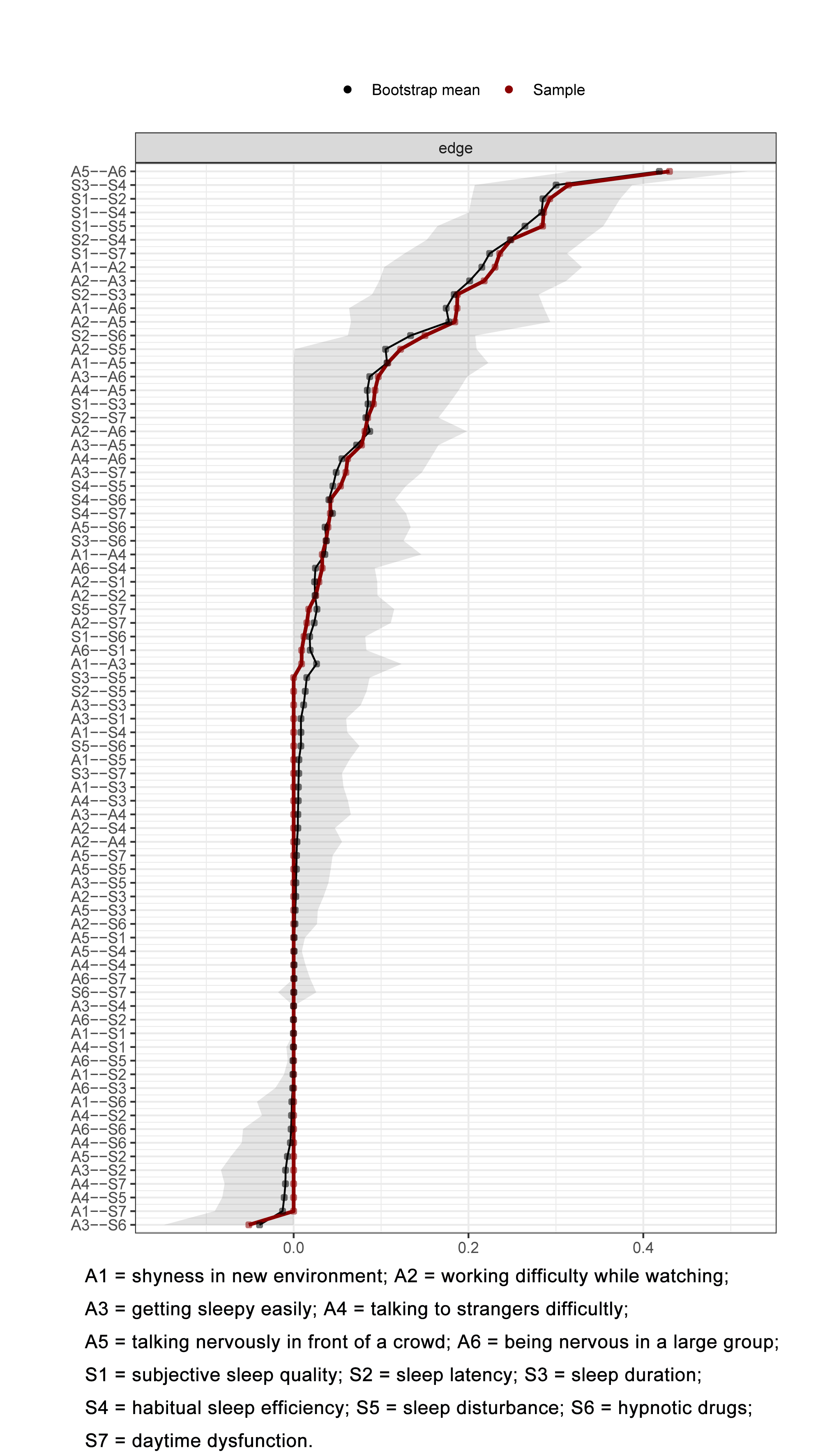

Supplement: Supplementary file 1 — Supplementary Material 1: Figure S1. Accuracy test of edge weights in the social anxiety-sleep quality network. Note: The gray area represents the bootstrapped confidence intervals and the red line represents the sample edge weight values. A1 = shyness in new environment; A2 = working difficulty while watching; A3 = getting sleepy easily; A4 = talking to strangers difficultly; A5 = talking nervously in front of a crowd; A6 = being nervous in a large group; S1 = subjective sleep quality; S2 = sleep latency; S3 = sleep duration; S4 = habitual sleep efficiency; S5 = sleep disturbance; S6 = hypnotic drugs; S7 = daytime dysfunction [file 12888_2023_5262_MOESM1_ESM.tif]

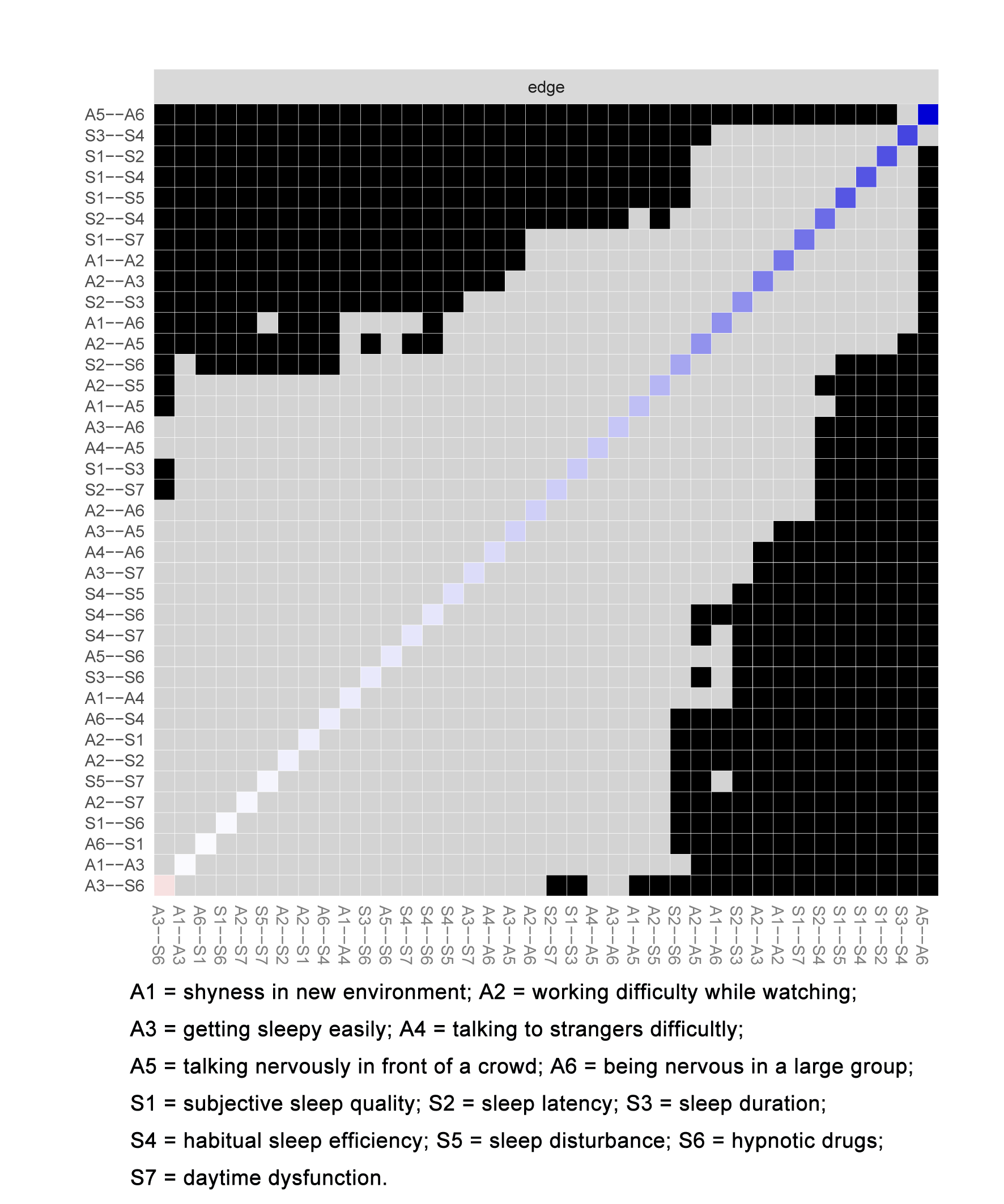

Supplement: Supplementary file 2 — Supplementary Material 2: Figure S2. Bootstrapped difference test of edge weights in the social anxiety-sleep quality network. Note: The gray box represents that the edge weights of the two corresponding node pairs have no significant difference, and the black box represents a significant difference. Blue and red boxes on the diagonal represent positive and negative edge weights, respectively. A1 = shyness in new environment; A2 = working difficulty while watching; A3 = getting sleepy easily; A4 = talking to strangers difficultly; A5 = talking nervously in front of a crowd; A6 = being nervous in a large group; S1 = subjective sleep quality; S2 = sleep latency; S3 = sleep duration; S4 = habitual sleep efficiency; S5 = sleep disturbance; S6 = hypnotic drugs; S7 = daytime dysfunction [file 12888_2023_5262_MOESM2_ESM.tif]

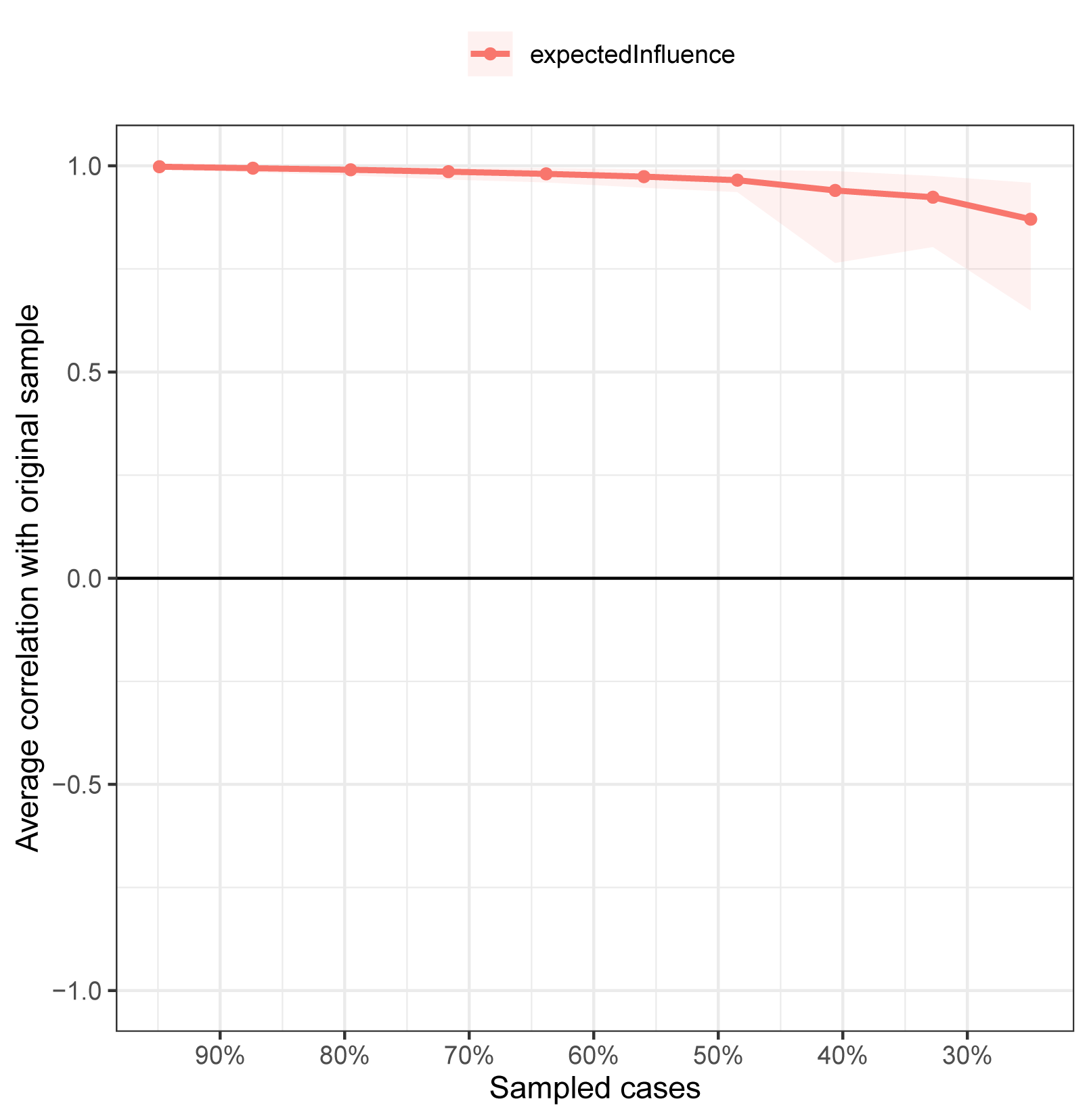

Supplement: Supplementary file 3 — Supplementary Material 3: Figure S3. Stability of expected influences in the social anxiety-sleep quality network. Note: The red bar represents the average correlation between expected influences in the full sample and subsample with the red area depicting the 2.5th quantile to the 97.5th quantile [file 12888_2023_5262_MOESM3_ESM.tif]

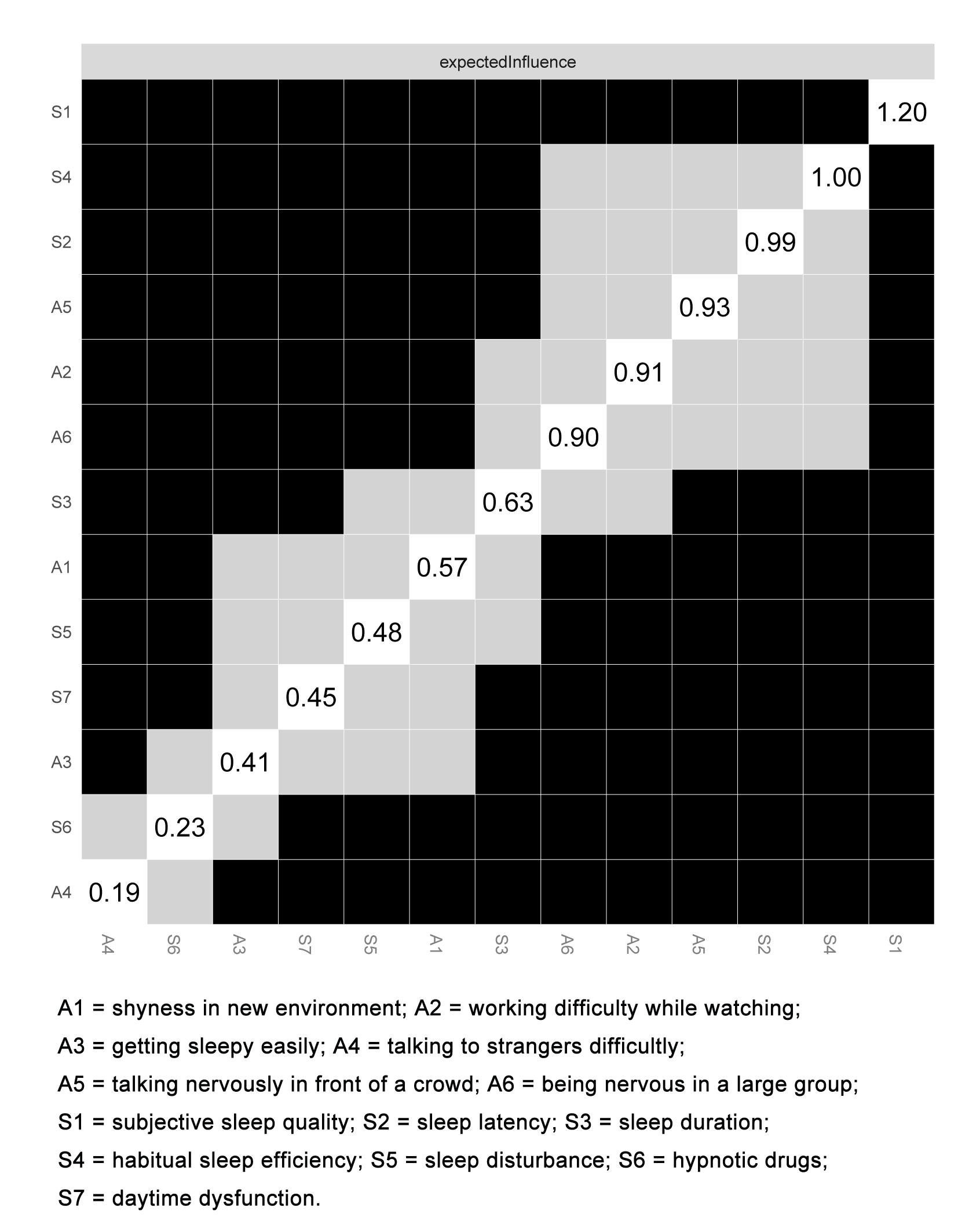

Supplement: Supplementary file 4 — Supplementary Material 4: Figure S4. Bootstrapped difference test of expected influences in the social anxiety-sleep quality network. Note: The black box indicates that the expected influences of the two corresponding nodes have a significant difference, the gray box indicated no significant difference. A1 = shyness in new environment; A2 = working difficulty while watching; A3 = getting sleepy easily; A4 = talking to strangers difficultly; A5 = talking nervously in front of a crowd; A6 = being nervous in a large group; S1 = subjective sleep quality; S2 = sleep latency; S3 = sleep duration; S4 = habitual sleep efficiency; S5 = sleep disturbance; S6 = hypnotic drugs; S7 = daytime dysfunction [file 12888_2023_5262_MOESM4_ESM.tif]

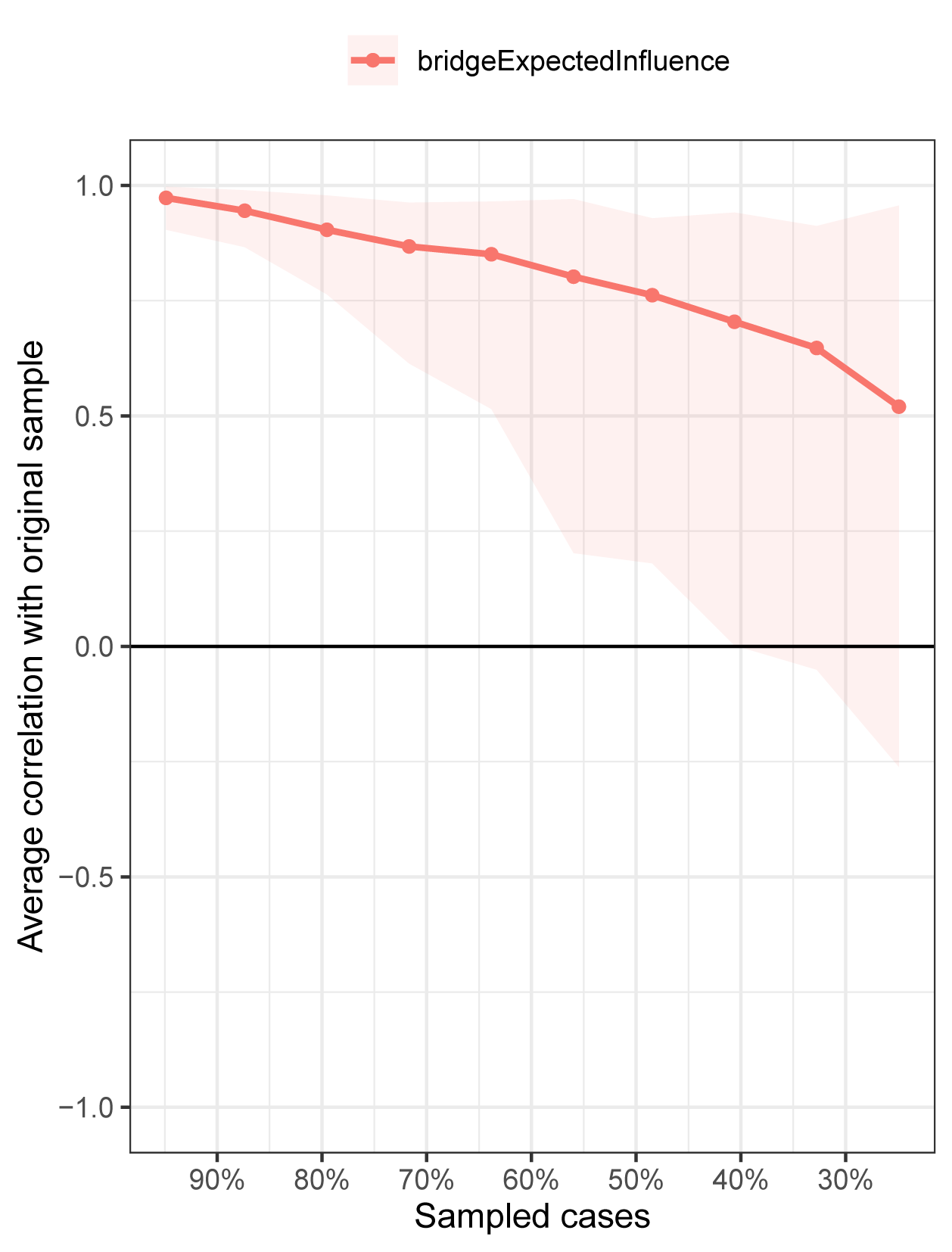

Supplement: Supplementary file 5 — Supplementary Material 5: Figure S5. Stability of bridge expected influences in the social anxiety-sleep quality network. Note: The red bar represents the average correlation between bridge expected influences in the full sample and subsample with the red area depicting the 2.5th quantile to the 97.5th quantile. [file 12888_2023_5262_MOESM5_ESM.tif]

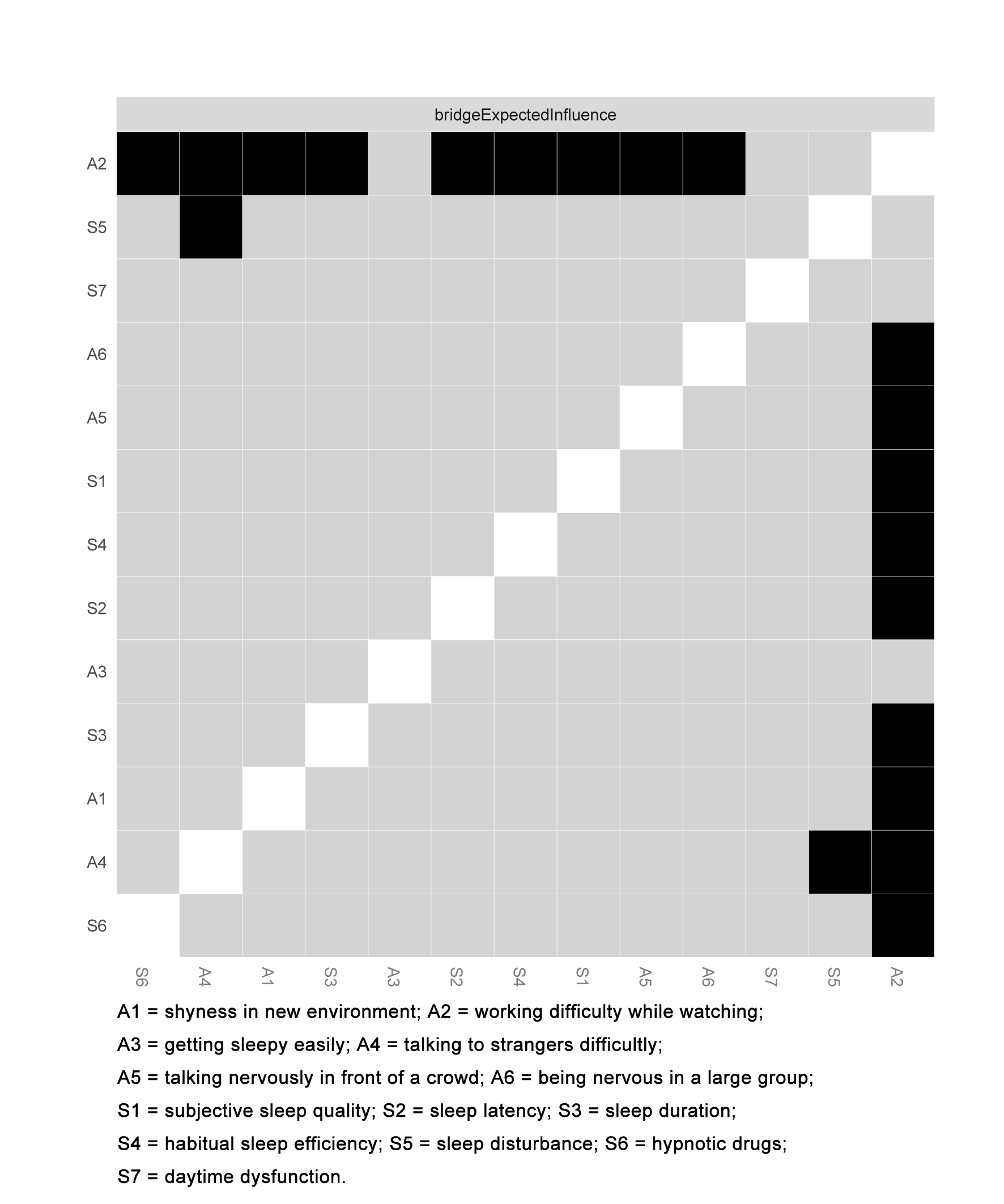

Supplement: Supplementary file 6 — Supplementary Material 6: Figure S6. Bootstrapped difference test of bridge expected influences in the social anxiety-sleep quality network. Note: The black box indicates that the bridge expected influences of the two corresponding nodes have a significant difference, the gray box indicated no significant difference. A1 = shyness in new environment; A2 = working difficulty while watching; A3 = getting sleepy easily; A4 = talking to strangers difficultly; A5 = talking nervously in front of a crowd; A6 = being nervous in a large group; S1 = subjective sleep quality; S2 = sleep latency; S3 = sleep duration; S4 = habitual sleep efficiency; S5 = sleep disturbance; S6 = hypnotic drugs; S7 = daytime dysfunction. [file 12888_2023_5262_MOESM6_ESM.tif]
