# Supplementary material for: Relationship of sleep-quality and social-anxiety in patients with breast cancer: a network analysis
Source: BMC Psychiatry. 2023 Nov 28;23:887. doi: 10.1186/s12888-023-05262-1 (PMC10683122; doi:10.1186/s12888-023-05262-1)
Supplement: Supplementary file 7 — Supplementary Material 7: Table S1. The correlation matrix in the social anxiety-sleep quality network of breast cancer patients [file 12888_2023_5262_MOESM7_ESM.docx]

Table S1 The [correlation](javascript:;) [matrix](javascript:;) in the social anxiety-sleep quality network of breast cancer patients

|  | A1 | A2 | A3 | A4 | A5 | A6 | S1 | S2 | S3 | S4 | S5 | S6 | S7 |
| --- | --- | --- | --- | --- | --- | --- | --- | --- | --- | --- | --- | --- | --- |
| A1 |  | 0.230 | 0.009 | 0.033 | 0.108 | 0.187 | 0.000 | 0.000 | 0.000 | 0.000 | 0.000 | 0.000 | 0.000 |
| A2 | 0.230 |  | 0.218 | 0.000 | 0.184 | 0.082 | 0.029 | 0.025 | 0.000 | 0.000 | 0.122 | 0.000 | 0.015 |
| A3 | 0.009 | 0.218 |  | 0.000 | 0.078 | 0.097 | 0.000 | 0.000 | 0.000 | 0.000 | 0.000 | -0.051 | 0.060 |
| A4 | 0.033 | 0.000 | 0.000 |  | 0.093 | 0.062 | 0.000 | 0.000 | 0.000 | 0.000 | 0.000 | 0.000 | 0.000 |
| A5 | 0.108 | 0.184 | 0.078 | 0.093 |  | 0.430 | 0.000 | 0.000 | 0.000 | 0.000 | 0.000 | 0.039 | 0.000 |
| A6 | 0.187 | 0.082 | 0.097 | 0.062 | 0.430 |  | 0.009 | 0.000 | 0.000 | 0.033 | 0.000 | 0.000 | 0.000 |
| S1 | 0.000 | 0.029 | 0.000 | 0.000 | 0.000 | 0.009 |  | 0.293 | 0.091 | 0.286 | 0.285 | 0.012 | 0.236 |
| S2 | 0.000 | 0.025 | 0.000 | 0.000 | 0.000 | 0.000 | 0.293 |  | 0.187 | 0.249 | 0.000 | 0.150 | 0.085 |
| S3 | 0.000 | 0.000 | 0.000 | 0.000 | 0.000 | 0.000 | 0.091 | 0.187 |  | 0.314 | 0.000 | 0.037 | 0.000 |
| S4 | 0.000 | 0.000 | 0.000 | 0.000 | 0.000 | 0.033 | 0.286 | 0.249 | 0.314 |  | 0.054 | 0.042 | 0.042 |
| S5 | 0.000 | 0.122 | 0.000 | 0.000 | 0.000 | 0.000 | 0.285 | 0.000 | 0.000 | 0.054 |  | 0.000 | 0.017 |
| S6 | 0.000 | 0.000 | -0.051 | 0.000 | 0.039 | 0.000 | 0.012 | 0.150 | 0.037 | 0.042 | 0.000 |  | 0.000 |
| S7 | 0.000 | 0.015 | 0.060 | 0.000 | 0.000 | 0.000 | 0.236 | 0.085 | 0.000 | 0.042 | 0.017 | 0.000 |  |
